# Moving towards the goals of FP2020 — classifying contraceptives^☆^

**DOI:** 10.1016/j.contraception.2016.05.015

**Published:** 2016-10

**Authors:** Mario Philip R. Festin, James Kiarie, Julie Solo, Jeffrey Spieler, Shawn Malarcher, Paul F.A. Van Look, Marleen Temmerman

**Affiliations:** a Department of Reproductive Health and Research, UNDP–UNFPA–UNICEF–WHO–World Bank Special Programme of Research, Development and Research Training in Human Reproduction (HRP), World Health Organization, Switzerland; b Independent consultant; c United States Agency for International Development (USAID), USA

## Abstract

With the renewed focus on family planning, a clear and transparent understanding is needed for the consistent classification of contraceptives, especially in the commonly used modern/traditional system. The World Health Organization Department of Reproductive Health and Research and the United States Agency for International Development (USAID) therefore convened a technical consultation in January 2015 to address issues related to classifying contraceptives.

The consultation defined modern contraceptive methods as having a sound basis in reproductive biology, a precise protocol for correct use and evidence of efficacy under various conditions based on appropriately designed studies. Methods in country programs like Fertility Awareness Based Methods [such as Standard Days Method (SDM) and TwoDay Method], Lactational Amenorrhea Method (LAM) and emergency contraception should be reported as modern. Herbs, charms and vaginal douching are not counted as contraceptive methods as they have no scientific basis in preventing pregnancy nor are in country programs. More research is needed on defining and measuring use of emergency contraceptive methods, to reflect their contribution to reducing unmet need.

The ideal contraceptive classification system should be simple, easy to use, clear and consistent, with greater parsimony. Measurement challenges remain but should not be the driving force to determine what methods are counted or reported as modern or not. Family planning programs should consider multiple attributes of contraceptive methods (e.g., level of effectiveness, need for program support, duration of labeled use, hormonal or nonhormonal) to ensure they provide a variety of methods to meet the needs of women and men.

## 1 Background

The global community came together in July 2012 at the London Summit on Family Planning and set the goal that an additional 120 million women and girls will have access to effective family planning (FP) information and services by the year 2020. With FP2020, commitments from national governments, civil society and the private sector were made to address the many supply and demand barriers that affect access and use of contraceptives. To monitor progress towards the goals, indicators on the use of modern and effective methods were adopted [1].

The most common classification system for contraceptives involves dividing methods into the categories of modern or traditional, but what are the modern methods of contraception? There remain inconsistencies in the definition and criteria for classifying methods as modern. Methods, such as the Lactational Amenorrhea Method (LAM) and Fertility Awareness Based Methods (FABMs) like the Standard Days Method (SDM) and the TwoDay Method, are classified as modern by some organizations and countries and as traditional by others. Other methods for avoiding pregnancy, such as emergency contraceptives (EC), while usually considered as a modern method, are currently not included in reports of contraceptive method use, in part due to difficulties in measuring and estimating coverage of use.

With the renewed focus on FP, it is critical that there is a clear understanding about the consistent and transparent classification of contraceptive methods and valid measures for determining contraceptive users. The World Health Organization Department of Reproductive Health and Research and United States Agency for International Development (USAID) convened a technical consultation in January 2015 to address issues related to classifying and reporting contraceptives. The consultation discussed the implications and approaches to contraceptive classification, measurements of contraceptive use and proposed revisions to existing approaches of classification, including the most often used modern versus traditional system of classification.

## 2 Implications of classification

The system of classifying contraceptives can influence calculations of key indicators, such as unmet need and contraception prevalence rate (CPR), that affect what counts towards reaching FP2020 goals. The way methods are categorized also influences investments to expand, strengthen or introduce other or new methods into programs. Ultimately, these investments influence provider behavior and client choice.

### 2.1 Unmet need and other indicators

Indicators impacted by any classification of contraceptive methods include CPR, unmet need for FP, contraceptive method mix, contraceptive discontinuation and others. Unmet need for FP is defined as the percentage of women who do not want to become pregnant [in the next 2 years] but are not using contraception. However, recent calculations of unmet need focused on unmet need for modern contraception, equating use of traditional methods with nonuse [2]. FP2020 also specified unmet need for a modern method, which differs from the usual definition of unmet need in that the former assumes that women using traditional methods have an unmet need for a more effective method [1]. The Guttmacher Institute's *Adding it up* report also focused on unmet need for modern contraception and, therefore, classifies users of traditional methods as having an unmet need [3]. Unmet need for modern contraception is an important measure for planning and advocacy. Some reports would count LAM and SDM users as having an unmet need even though these methods have been found to be highly effective when used correctly and are preferred by some women. In other cases, these same LAM and SDM users would be counted as having a met need making these two calculations noncomparable.

### 2.2 Introduction of methods into programs and client choice

“Modern methods” are often believed to be more effective than “traditional methods”. Program managers and decision makers thus prefer to invest in supporting provision of “modern methods”. When even those methods that can be highly effective are classified as “traditional”, program managers and providers may be less likely to consider including them in programs. This ultimately affects what methods a client can choose from and what would be offered at their local clinic or pharmacy. Having a wide range of methods available allows women and men to select a contraceptive that best fits their lifestyle and need. A strong program also ensures accurate information so that women and men truly understand the relative effectiveness, mode of action and side effects of different methods and they can make an informed choice.

## 3 Approaches to contraceptive classification

The dichotomy modern/traditional is the most commonly used classification for contraceptives, but there are other systems of classification described below and presented in Table 1.

### 3.1 Modern versus traditional

Part of the challenge for any classification system is the lack of a clear consensus on definitions and criteria for the categories of modern and traditional. None of the definitions considered at the consultation was found to be consistent when applied to the current range of available contraceptive methods. Present use of *modern* and *traditional* does not correspond to the temporal or historical context of these words. Condoms have existed for hundreds of years yet are considered as modern [4]. Modern and traditional are also not consistently applied to denote more and less effective contraceptive methods. Withdrawal, generally considered a traditional method, is as effective as the condom [5], [6]. An underlying unspoken interpretation of these terms is the value judgment that modern means “good” and traditional means “bad”. The unintended consequences of the current classification system are that country FP programs support “modern” methods as being more effective and discourage use of “traditional” methods as less effective.

A recent commentary by Hubacher and Trussell proposed to define a modern contraceptive method as a product or medical procedure that interferes with reproduction from acts of sexual intercourse [7]. They defined all FABMs and LAM as nonmodern methods, which would have major implications for the many programs that actively promote these methods in countries.

In the January 2015 consultation, it was agreed that a modern contraceptive method should have the following characteristics: a sound basis in reproductive biology, a precise protocol for correct use and existing data showing that the method has been tested in an appropriately designed study to assess efficacy under various conditions. With these characteristics, new contraceptive methods when they come on the market would generally be included as modern. All new contraceptive innovations should be tested against these criteria in order to be defined as “modern”.

In order to work towards the FP2020 goals, the FP community needs greater consistency, clarity and transparency in classifying contraceptives. Using the terms modern and traditional only does not fully meet these requirements.

### 3.2 Other classification systems

Some of the more familiar method classification subcategories include permanent methods, long-acting reversible contraception, temporary methods and multipurpose prevention methods. The World Health Organization (WHO) analysis of quantitative indicators on human rights within contraceptive programs describes as a measure of contraceptive method mix the proportion of facilities that provide a range of methods that meet women's needs, including at least one short-acting, one long-acting, one permanent and one emergency method [8].

In order for countries to be responsive to the needs of their citizens, FP programs should consider multiple attributes of contraceptive methods (e.g., level of effectiveness, need for program support, duration of labeled use, hormonal or nonhormonal) to ensure they provide a variety of methods to meet the needs of women and men.

Table 1, prepared from the discussions during the consultation and using the information from *Family planning: a global handbook for providers*, summarizes where methods should be classified based on various characteristics and could be the basis for decisions by country programs [5], [6].

### 3.3 Methods that are not consistently classified

Some methods are classified differently by various agencies or surveys. Different surveys report LAM and breastfeeding as separate methods and inconsistently as either modern or traditional. LAM is reported as a modern method in Demographic and Health Surveys (DHS) [9], [10] and as traditional in Multiple Indicator Cluster Surveys [11]. The United Nations Department of Economic and Social Affairs reports breastfeeding and LAM together as part of other traditional methods, which also include periodic abstinence, douching and various folk methods. [12].

In DHS, an average of 0.8% of all women respondents per survey reported current LAM use [9]. Among self-reported LAM users, only 26% met the correct-practice criteria [13]. It is advised to enhance interviewer training to ensure that LAM (as defined by its criteria) and breastfeeding (not necessarily intended for use as a contraceptive method) are separately and appropriately coded during data collection. LAM has its own defined criteria for use, which includes the intent of using breastfeeding for its contraceptive effect. Present surveys should continue to identify this intent through specific questions to define a LAM user. In regions where LAM is promoted, taught and used, it should be reported as a modern method.

EC pills, both levonorgestrel and ulipristal acetate, are generally considered as modern methods, and they fulfill our definition of a modern method. Studies have documented its effectiveness in preventing pregnancy. However, it has been difficult methodologically to quantify EC use. Social marketing organizations and USAID count 20 packs of EC sold/distributed as being equivalent to one Couple-Year of Protection. PMA2020 (Performance Monitoring and Accountability 2020) counts anyone who reports current EC use or has used EC as her most recent method within the past 12 months as a current EC user [14]. Similarly, the impact of EC use has been difficult to establish. For instance, its effectiveness in lowering abortion rates at the population level has not yet been demonstrated [15]. Further work is needed to develop appropriate methodologies for measuring and reporting EC use and impact as part of regular FP indicators.

For contraceptive methods that are not yet regularly or commonly reported but are actively included in country programs, it is important that reporting agencies be informed about which countries have programs promoting these specific methods, mentioning the standard criteria used for measurement. What should be included as modern methods are FABMs, such as the SDM, and the TwoDay Method, which are more effective than the traditional calendar/rhythm method. Data on their use should be collected and reported separately in countries where these methods are promoted in FP programs.

There is efficacy evidence for both SDM [16] and the TwoDay Method [17], showing these methods as more effective than the calendar/rhythm method. These studies were designed to include a 3-month learning phase for participants, of which over 90% moved to the 12-month efficacy phase. More efficacy studies are always desired, especially in various circumstances. The available efficacy studies were, however, considered by many in the consultation as adequate, properly designed and with enough power, as basis for inclusion as a modern method. The SDM study covered three countries and included various types of users who used SDM. Counseling was included as part of the provided services, which are standard for FABMs and all other contraceptive methods. Pregnancies were measured in both learning and efficacy phases, with typical and perfect rates estimated for the latter [18]. A report by Sinai et al. also showed decreasing annual trends in typical use life-table pregnancy rates per year in using SDM from efficacy studies and method instruction studies [19].

Although this evidence only includes a limited number of studies, there are also reports on significant global programmatic experience showing that these methods are important options in the method mix: SDM in the USA and over 22 countries in Africa, Asia and Latin American and the TwoDay Method in the USA, Guatemala, Peru, Democratic Republic of Congo, Rwanda and Uganda. The other FABM methods, such as the Billings and Sympto-thermal methods, were not discussed during the consultation.

Withdrawal is commonly practiced in many countries to varying degrees of effectiveness and the consultation agreed that it should continue to be classified as traditional, especially since it is not included among methods actively promoted in programs.

Herbs, charms, folk methods and vaginal douching do not have any scientific basis as being effective in preventing pregnancy, so these should not be included nor classified as contraceptive methods. Thus, if a woman reports use of any of these in response to a question about her contraceptive use, the interviewer should record “None”. No FP program is promoting these methods.

## 4 Proposals for using existing contraceptive classifications

Policies in FP should promote provision of a wide range of effective methods from which women and men can choose. However, current classification systems create challenges since some effective FABMs and EC are inconsistently categorized. Using only the category system with modern and traditional as basis for planning and programming is inadequate and confusing. While there was an attempt to clarify the dichotomy of classifying as modern or traditional, the conclusions from the meeting also state that using other classification criteria or systems may be more useful for specific purposes, as presented in Table 1.

LAM and the various FABMs should continue to be measured and reported in international surveys conducted by agencies. Participants need to be asked not only about what method was used but also the components of some methods, such as LAM, to validate its defined use. Reports that group methods such as “fertility awareness", “short acting", “long acting", and other terms are inconsistently applied. This causes confusion, does not allow for comparative analysis, and loses the specificity of information critical for decision makers. Reports should include method-specific results. This results in combining methods that are either considered modern (e.g., SDM or TwoDay Method) or traditional (e.g., calendar method), even if these should be separately reported.

It is important that WHO identifies a classification system that is evidence based and facilitates selection of a wide range of methods that are effective and acceptable to the norms and standards of clients. The classification system should be guided by the goals of improving access to effective contraceptive methods and supporting the reproductive rights of women and men. Measurement challenges will need to be addressed, but they should not be the driving force to determine what methods are counted and which are not. The WHO document *Ensuring human rights within contraceptive programs: a human rights analysis of existing quantitative indicators* provides a systematic, transparent system that is required to explicitly link health concerns and human rights [8]. Accountability is central to ensuring that health and human rights standards are respected, protected and fulfilled. In accordance with human rights principles, people should have access to the widest range of contraceptive methods from which to choose to meet their needs and preferences and their changing needs throughout their reproductive lives.

An ideal classification system for contraceptive methods should be simple and parsimonious, should lead to greater clarity and consistency and should be easy to use and understand by a broad set of stakeholders including researchers, program managers, policymakers and other potential users. However, any changes should not be overly disruptive to present systems of data reporting or jeopardize the ability to evaluate trends. As a first step, there is need for consistency across reporting systems using clear and well-defined criteria when classifying contraceptives by the most frequently used terms of modern and traditional (Table 1).

## Figures and Tables

**Table 1:** Table of classification systems of contraceptive methods

Classification Systems of Contraceptive Methods	Female Sterilization	Vasectomy	Implant	Copper Intrauterine Device	Hormonal Intrauterine System	Injectable	Oral Contraceptive Pill	EC Pill, 1.5 mg Levonorgestrel	Male Condom	Female Condom	Diaphragm	LAM	SDM	Two Day Method	Rhythm/Calendar Method	Withdrawal
**Modern or traditional**	M	M	M	M	M	M	M	M	M	M	M	M	M	M	T	T
**Level or tier of effectiveness**	1	1	1	1	1	2	2	3^a^	3	3	3	2	3	3	4	4
**Need for program support**	Hi	Hi	Hi	Hi	Hi	Me	Lo	Lo	Lo	Lo	Lo	Me	Me	Me	No	No
**Duration of labeled use**	P	P	LA	LA	LA	MA	SA	SA	SA	SA	SA	MA	SA	SA	SA	SA
**Male or female controlled, or both**	FC	MC	FC	FC	FC	FC	FC	FC	MC	FC	FC	FC	Both	Both	Both	MC
**Coitally dependent/related**	N	N	N	N	N	N	N	Y	Y	Y	Y	N	Y	Y	Y	Y
**Need for surgical procedure to use**	Y	Y	Y	N	N	N	N	N	N	N	N	N	N	N	N	N
**Presence of hormones**	N	N	Y	N	Y	Y	Y	Y	N	N	N	N	N	N	N	N
**Client's ability to discontinue without needing a provider**	N	N	N	N	N	Y	Y	Y	Y	Y	Y	Y	Y	Y	Y	Y
**Return to fertility after discontinuation of method**	U	U	I	I	I	D	I	I	I	I	I	D	I	I	I	I

***Legend to Table:***																
*Modern and traditional*			*M — Modern, T — Traditional*													
*Level or tier of effectiveness*			*1, 2, 3 or 4 (as per Family Planning, Global Handbook for Providers, 2011 update)*													
*Need for program support*			*Hi — High, Me — Medium, Lo — Low, No — None*					*High — requires a clinic setting with trained and skilled providers*								
								*Medium — can be provided in a nonclinical setting by trained and skilled providers*								
								*Low — can be provided in community distribution programs, over the counter or in informal settings*								
*Duration of labeled use*			*P — Permanent, LA — Long-acting, MA — Medium-acting, SA — Short-acting*					*Note: Medium-acting is not a commonly used category but is presented here to distinguish it from the methods that are labeled for a lesser period of effect.*								
*Male or female controlled, or both*			*MC — Male controlled, FC — Female controlled, Both*													
*Coitally dependent/related*			*Y — Yes, N — No*					*Coitally dependent: Requires a specific intervention at the time of intercourse.*								
*Need for surgical procedure to use*			*Y — Yes, N — No*													
*Presence of hormones*			*Y — Yes, N — No*													
*Client's ability to discontinue without reliance on a provider*			*Y — Yes, N — No*													
*Return to fertility after discontinuation of method*			*I — Immediate, D — Delayed, Ne — Never, U — Uncertain success after reversal*													

Note: This table shows the various classification systems, in addition to modern and traditional, for the most commonly used contraceptive methods.

a If 100 women used progestin-only EC pill, one would likely become pregnant (from Family Planning, Global Handbook for Providers, 2011 update). Please note that effectiveness in EC pill studies was computed on women use after one act of protected intercourse, which would be different from analyses of other contraceptive effectiveness studies.

## Appendix A Supplementary data

List of Participants Image 1
