# Supplementary material for: Moving towards the goals of FP2020 — classifying contraceptives
Source: Contraception. 2016 Oct;94(4):289–94. doi: 10.1016/j.contraception.2016.05.015 (PMC5032916; doi:10.1016/j.contraception.2016.05.015)
Supplement: Supplementary file 1 — List of Participants [file mmc1.docx]

**Supplemental Table**

WHO/USAID Technical Consultation on Classification of Contraceptive Methods

WHO Headquarters, 20-21 January 2015

Disclaimer: The views expressed do not necessarily represent the views of all the participants and the institutions of the authors of this Commentary.

Technical consultation participants

Katherine Ba-Thike, ***C***onsultant in Sexual and Reproductive Health

Jacqueline Darroch, Guttmacher Institute

Karen Hardee, The Evidence Project, Population Council

Roy Jacobstein, IntraHealth

Victoria Jennings, Institute for Reproductive Health (IRH), Georgetown University

Jeff Jordan, Population Research Bureau

Shawn Malarcher, USAID

Nina Miller, Family Planning 2020

Nuriye Ortayli, UNFPA

Scott Radloff, The Johns Hopkins University, Bloomberg School & Public Health

Hantamalala Rafalimanana, United Nations Population Division

Kia Reinis, Demographic &Health Surveys (DHS) Program, ICF International

Jose Rimon II, Bill & Melinda Gates Institute for Population and Reproductive Health

Madeleine Short Fabic, USAID

Emily Sonneveldt, Director Track20 Project Futures Institute

Markus Steiner, FHI 360

# Julie Solo, Consultant in Sexual and Reproductive Health

# Jeff Spieler, Consultant in Sexual and Reproductive Health

# James Trussell, Princeton University Princeton NJ 08544 United States of America

## Paul Van Look, Consultant in Sexual and Reproductive Health

## Elizabeth Westley, International Consortium for Emergency Contraception

# WHO Secretariat:

## James Kiarie

Mario Festin

Moazzam Ali

Asa Cuzin-Kihl

Mary Lyn Gaffield

Rajat Khosla

## Gunta Lazdane

Suzanne Reier

## Lale Say

## Suzanne Jacob Serruya

Petrus Steyn
